# Use of folic acid supplementation to halt and even reverse the progression of gastric precancerous conditions: a meta-analysis

**DOI:** 10.1186/s12876-022-02390-y

**Published:** 2022-08-02

**Authors:** Jing Lei, Fugang Ren, Wenyuan Li, Xiaochuan Guo, Qingsong Liu, Hongjing Gao, Yaobin Pang, Yingjie He, Jing Guo, Jinhao Zeng

**Affiliations:** 1 Dermatological Department, Hospital of Chengdu University of Traditional Chinese Medicine and Chengdu University of Traditional Chinese Medicine, 37 Shierqiao Road, Chengdu, Sichuan 610000 People’s Republic of China; 2 College of Medical Technology, Chengdu University of Traditional Chinese Medicine, Chengdu, People’s Republic of China; 3 Sichuan Evidence-Based Medicine Center of Traditional Chinese Medicine, Hospital of Chengdu University of Traditional Chinese Medicine, Chengdu, People’s Republic of China; 4 Geriatric Department, Hospital of Chengdu University of Traditional Chinese Medicine, 39 Shi-er-qiao Road, Chengdu, Sichuan 610072 People’s Republic of China; 5 TCM Regulating Metabolic Diseases Key Laboratory of Sichuan Province, Hospital of Chengdu University of Traditional Chinese Medicine, Chengdu, Sichuan 610072 People’s Republic of China

**Keywords:** Folic acid, Gastric cancer, Meta-analysis, Gastric precancerous conditions, Chronic atrophic gastritis, Intestinal metaplasia, Dysplasia, Precancerous lesions

## Abstract

**Background:**

Current data indicate that supplements such as folic acid and vitamin B may be beneficial in halting and even reversing atrophic gastritis, intestinal metaplasia and intraepithelial neoplasia, generally referred to as gastric precancerous conditions(GPC). However, there is no Meta-analysis article to evaluate the prevention and treatment of folic acid in the gastric precancerous conditions. We therefore conducted a meta-analysis to confirm the efficacy of folic acid in treating GPC.

**Methods:**

Using a systematic review method, consider randomized controlled trials (RCT), including clinical trial reports, unpublished clinical trial data, and conference papers. The search time was been set from the database’s establishment to June 2, 2021. The language was not limited, using PubMed, SinoMed, Lancet, Web of Science, CNKI, Cochrane, Ovid, Science Direct, Embase, and EBSCO databases. Data were extracted using a pre-designed extraction tool and analysis was undertaken using RevMan5.2.Besides,we use Origin software to construct the Time-dose interval analysis.

**Results:**

Of the 225 records identified, 13 studies involving 1252 patients (including 11 clinical controlled trials, 1 conference paper report and 1 unpublished research report) met the inclusion conditions. Folic acid dose maintained at 20–30 mg* / d* for 3–6 months may be beneficial to pathological changes of GPC. Moreover, in the 3 month treatment of 5 trials, the effect was more obvious when the folic acid dose was maintained at 30 mg* / d*. In the 7 trials, the symptom ineffective rate of GPC treated with folic acid was 32% (*RR:0.32, 95%* confidence interval *CI:0.21–0.48),* which was combined using a fixed analysis model; The effect of folic acid on gastric mucosal atrophy in 5 trials *(RR: 1.61, 95%CI 1.07–2.41)*. The changes of folic acid on intestinal metaplasia in the 2 experiments (*RR: 1.77, 95% CI: 1.32–2.37)*.The 2 results are combined using a fixed analytical model. However, the subgroup analysis of 9 trials revealed no significant effectiveness of symptom.

**Conclusions:**

Our research showed that folic acid supplementation brings benefits in preventing and even reversing the progression of GPC in the stomach, and provided evidence for its potential clinical use in management of GPC.

***Registration:*** The logn number of our Meta-anlysis on PROSPERO is CRD420223062.

**Supplementary Information:**

The online version contains supplementary material available at 10.1186/s12876-022-02390-y.

## Background

Gastric precancerous conditions (GPC) are closely related to gastric cancer. Chronic atrophic gastritis and intestinal metaplasia are considered to be precancerous lesions. They can not only independently lead to the occurrence of gastric cancer, but also further constitute dysplasia and adenocarcinoma [1–6]. Therefore, halting and even reversing chronic atrophic gastritis plays an important role of prevention and treatment of stomach cancer. Helicobacter pylori infection is a vital factor in the carcinogenesis of stomach mucosa in chronic atrophic gastritis [7], which can change the secretion of pepsinogen and gastrin and result in abnormal secretion of hydrochloric acid by parietal cells. These changes produce abnormal methylation [8–11] of the DNA associated with cancerous changes that eventually occur in stomach tumors as the condition worsens.

Folic acid is a component of human bodies that controls DNA synthesis, maintains DNA stability and integrity, and repairs abnormal DNA methylation and aberrations [12–14]. when patients with GPC were treated with folic acid, the apoptotic rate of gastric mucosal epithelial cells and the expression of tumor suppressor gene P53 were significantly increased, while the expression of certain oncogenic proteins was reduced to prevent further abnormal DNA methylation [15, 16]. Folic acid may bring benefits to the prevention and treatment of GPC.

At present, studies have confirmed that folic acid has a certain therapeutic effect on gastric precancerous conditions, but there is still a lack of systematic reviews. Consequently we carried out a prospective meta-analysis to understand the prevention and treatment effects of folic acid on GPC in the stomach. In the present study, we collected clinical studies on the use of folic acid in the treatment of GPC in various cities over the past 20 years, and analyzed its curative effect and provided guidance for gastric cancer prevention and treatment.

## Methods

### Search strategy

Our research was reported using the Preferred Reporting Items for Systematic Reviews and Meta-Analyses detailed list (PRISMA)[17]. We registered our research on PROSPERO with the registration number is CRD420223062.We conducted a comprehensive search by searching PubMed, SinoMed, Lancet, Web of Science, CNKI, Cochrane, Ovid, Science Direct, Embase, and EBSCO. Search terms were limited to “gastric precanserous conditions”, “Chronic atrophic gastritis”, ”folic acid” or “folate”, and “randomized controlled trials”. At the same time, we added “vitamin” as a text word. Types of articles searched include journal articles and grey literature. These articles had no language restrictions. The search time is set from the establishment of the database to June 2, 2021.

### Selection criteria

#### Inclusion criteria

(1) Patients with gastric precancerous cancer, Age > 18 years old; (2) Randomized controlled trials; (3) Research reported cured rate, efficiency, invalid, and pathological changes with a clearly definitions.(4) Intervention methods including folic acid supplementation (with or without vitamin B supplementation).

#### Exclusion criteria

(1) Studies using the same population or overlapping databases; (2) Participants in the study have other underlying diseases; (3) Disease diagnosis does not match the guidelines(We used the MAPS II guideline (2019) [18] as guidance); (4)Statistical method is wrong and the error cannot be corrected.

Folic acid was selected as the treatment method in this study, compared with other treatment methods such as combination therapy and placebo. The effective rate of clinical treatment or the reversal of pathologic changes was regarded as one of the key endpoints of the report.

### Literature quality evaluation

The Cochrane Collaboration tool was used by 2 authors(JL,WYL) to evaluated the quality of the studies independently [19, 20]. Seven specific domains was addressed in Cochrane Collaboration tool, namely: random sequence generation; incomplete outcome data; allocation concealment; blinding of outcome assessment; blinding of participants and personal; selective reporting; and other issues. The blinding and integrity of the outcome data for each outcome were evaluated by 2 authors separately.

We used standardized data extraction forms to collect information of published reports from Included studies. When necessary, we contacted the authors for additional information.

### Data collection

Two researchers (JL,WYL) independently reviewed the content of 225 abstracts to determine whether they met the eligibility criteria for inclusion. When there was a difference, the third investigator(Dr.Zeng)performed an additional evaluation. The data of all retrieved articles were extracted using the following headings and listed as a table: first author and years, types of gastric precancerous diseases, pathological changes indicators, intervention duration, course of disease, the sex and numbers of each group, mean age, and interventions.

### Data analysis

We extracted and analyzed the data of folic acid treatment for the clinical treatment of GPC, including efficiency, recurrence rate, pathological changes(including gastric mucosal atrophy, intestinal metaplasia, and intraepithelial neoplasia) for meta-analysis. After we extracted the main findings from the search results, Revman5.2 was used for meta-analysis and statistical synthesis [21]. We calculated the Relative Risk*(RR)* of dichotomy results and the Mean Difference *(MD)* of continuous results, and the Confidence Interval*(CI)* was set to 95%.When the result shows that *I*^*2*^ < 50% and *P* > 0.5, it indicates that has low heterogeneity, and the fixed effects model is employed. If *I*^*2*^ > 50% and *P* < 0.5, has high test heterogeneity, and the random effects model is employed. When the results are similar, the random effect model is preferred, because some researchers believe that the random-effect model is a more natural choice than the fixed effect model in medical decision-making [19, 22]. The fixed‐effect model and *95% CI* [23, 24] was used to analysis the results of the comparative tests. The heterogeneity of the study was assessed using Cochrane’s Q test (with *P* ≤ 0.05 as significant) and calculating quantified using the *I*^2^ statistic [20]. Moreover, the Time-dose interval analysis is constructed by Origin software to explore the duration of treatment and dosage of drugs for diseases.

## Results

### Overview of all contained studies

**Fig. 1:**
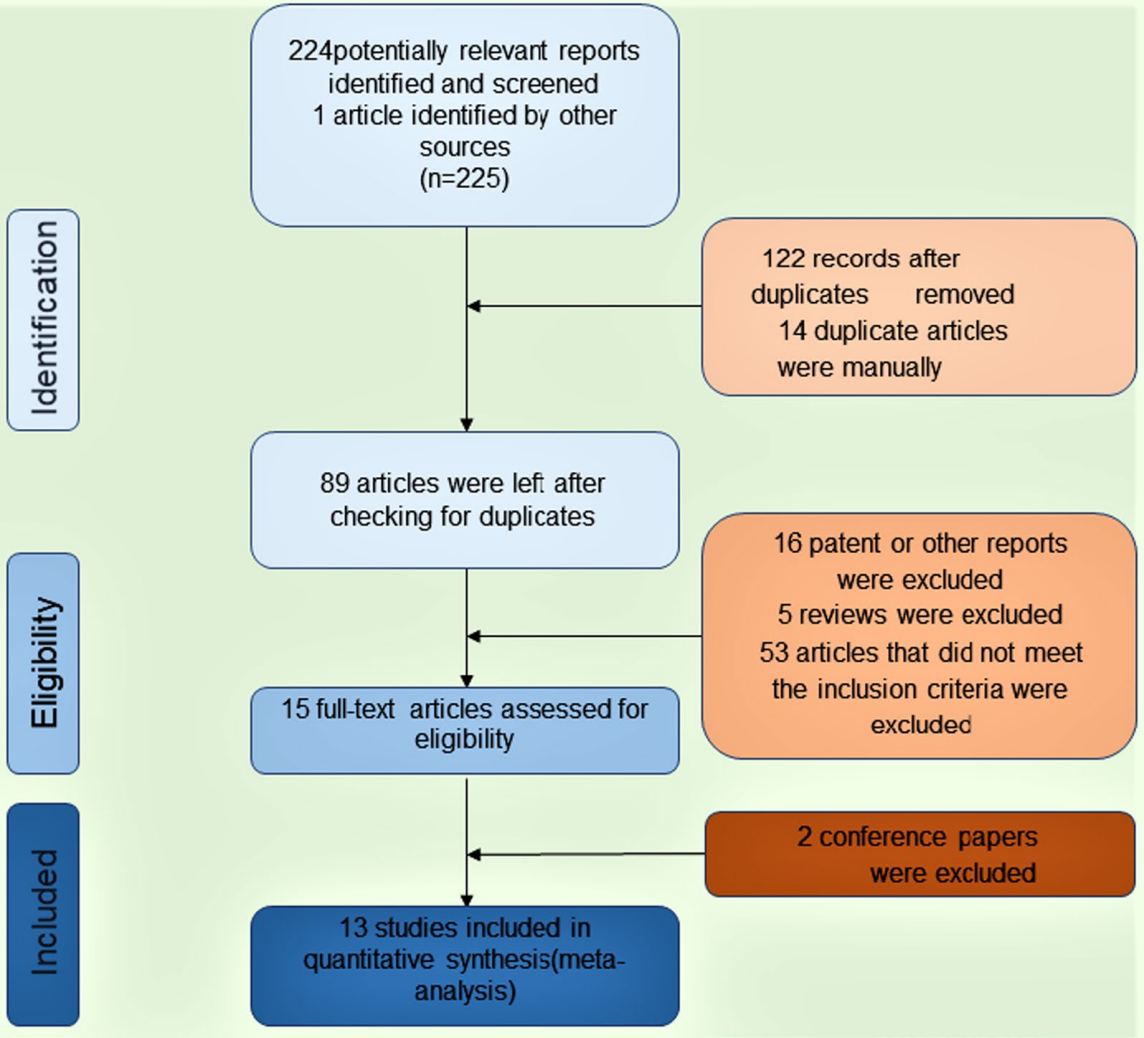
PRISMA flow diagram for study selection

The article flow of this review outlines in Fig. 1. In evaluating the 15 research trials, 2 conference papers were excluded due to no available data for analysis. We initially sought out various articles in English-related databases, but found that there were no publications on the prevention and treatment of folate-related GPC Meta-analysis. We ultimately analyzed 13 randomized controlled trials; 1 was an unpublished research result (JHZ), one was a conference article, so these 2 trials were used as gray data. And the remaining 11 were clinical randomized controlled trial reports (Fig. 1). Based on data from 13 trials [25–36], we evaluated the effectiveness of folic acid in treating GPC and even preventing gastric cancer. 2 of the studies used folic acid as the control group, and the remaining 11 studies used folic acid as the treatment group. We here included a total of 1252 individuals (the datas which used in our article is include in Additional file 1) [25–36].

### Clinical efficacy evaluation criteria [5, 6, 18, 37]

Cured means disappearance of clinical symptoms, disappearance of glands atrophy, and gastric mucosal return to normal; efficiency means disappearance or mitigate of clinical symptoms, reduced vascular stria the atrophy is reduced; invalid means that the clinical symptoms are not improved or worsened, and the gland atrophy has not changed; pathological alter include stomach mucosa atrophy and intestinal metaplasia.

### The study characteristics

Additional file 3: Table 1 was listed the baseline characteristics of all contained researches, including the first author, publication year, types of precancerous diseases included in the study, and outcome indicators. Additional file 2: Table 2 shows the characteristics of all participating researchers, including the patient's age and sex, course of disease, intervention measures, and intervention duration. The 13 research trials were all carried out in China, and the participants were all Chinese; the GPC observed by all researchers met the definition of chronic atrophic gastritis in the Chinese clinical diagnostic guidelines. The age, sex and length of illness of patients exerted no influence on the experiment.

### Risk of bias

**Fig. 2:**
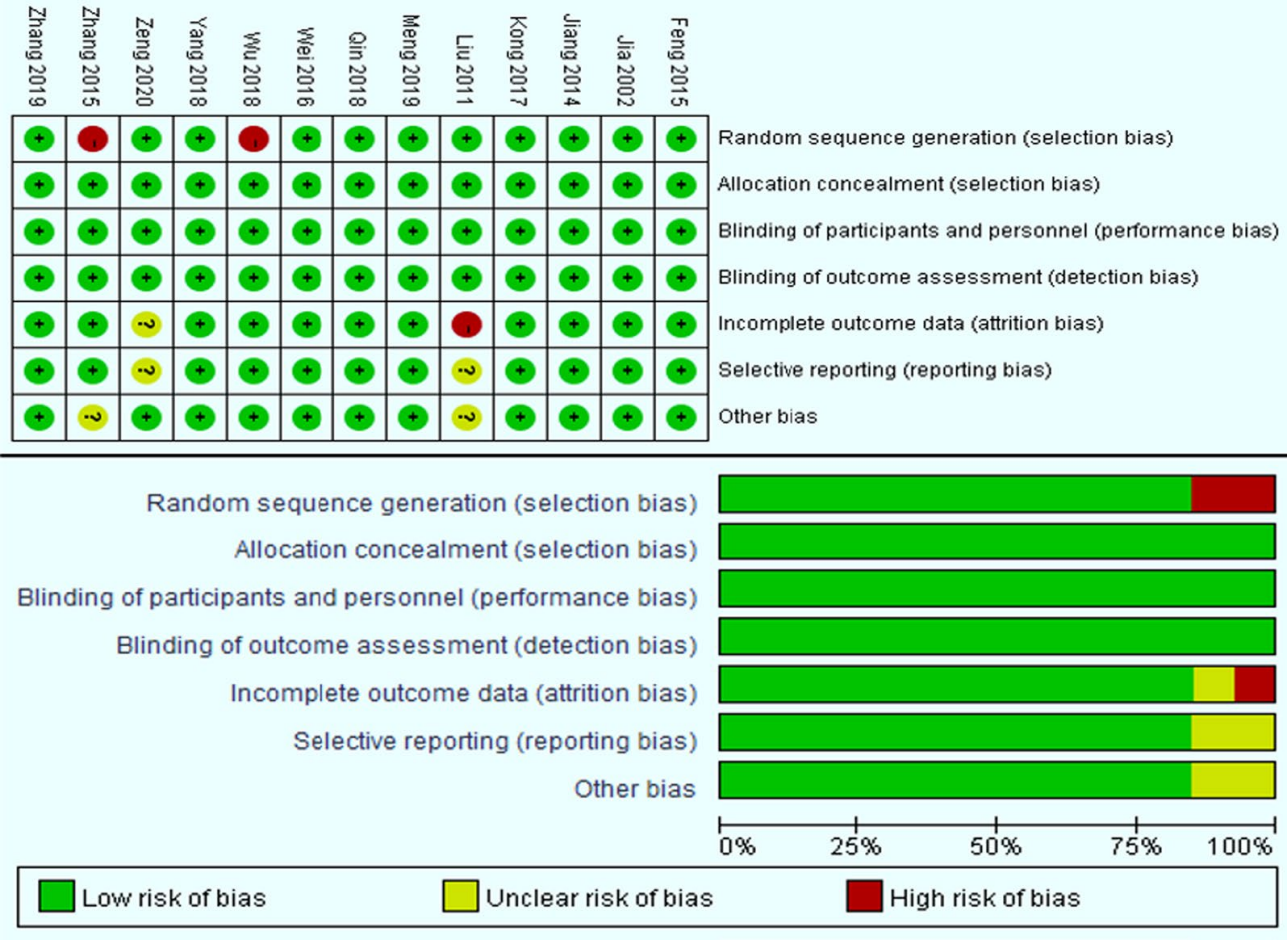
Risk of bias summary

The risk of bias in the included studies is summarized in Fig. 2. 2 authors independently estimated the risk of bias of all contained studies with the Cochrane tool, and any differences will be resolved through negotiation.

### Selection bias

11 studies [20–22,24-27,29–31], and an unpublished study written by Dr. Zeng] because it describes the use of sturdy random methods to generate random sequences(such as use of computer random number generator), the selection bias is low. The remaining two studies [28, 33] had a high risk of selection bias because the random sequence generation method was not mentioned.

### Allocation concealment

All studies mentioned the method of hiding the allocation to the study group, so all studies had a low risk of bias in this field.

### Detection bias: blinding of outcome assessment

The evaluators of all included studies independently evaluated the results of the study, so the had a low risk of detecting bias.

### Performance bias: blinding participants and personnel

The participants and researchers in all studies were double-blind, thus all studies had a low risk of bias of performance bias.

### Attrition bias: incomplete outcome data

The attrition bias of 11 studies is low risk of bias [25–35]. 1 study [36] has obvious distribution of the number of study groups and control groups The difference is therefore high risk. The results of 1 study reported that there was sample loss (an unpublishe research written by Dr. Zeng), so there was a relatively high risk of bias.

### Reporting bias: selective reporting

11 studies reported all the research results mentioned in the article [25–35], so they all have a low risk of bias. [31, and an unpublished research written by Dr. Zeng] 2 studies do not include all of the results, therefore relatively higher risk of bias.

### Other bias

Since without any other latent sources of bias was found, the 11 [25–32, 34, 35] studies were all had a low risk of bias in this filed. The remaining 2 studies [33, 36], owing to different research methods from other studies, have a relatively high risk of bias.

### Comparisons and outcomes

#### Comparisons and outcomes1

The effectiveness of Folic Acid for GPC symptom relief.

**Fig. 3:**
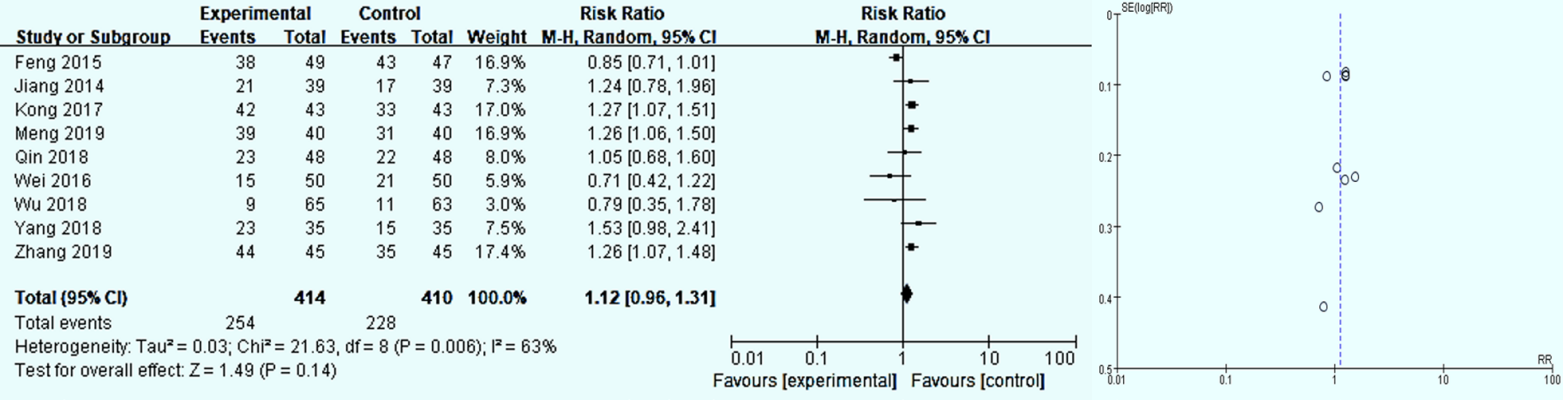
Analysis of the effectiveness of folic acid for GPC symptom relief

**Fig. 4:**
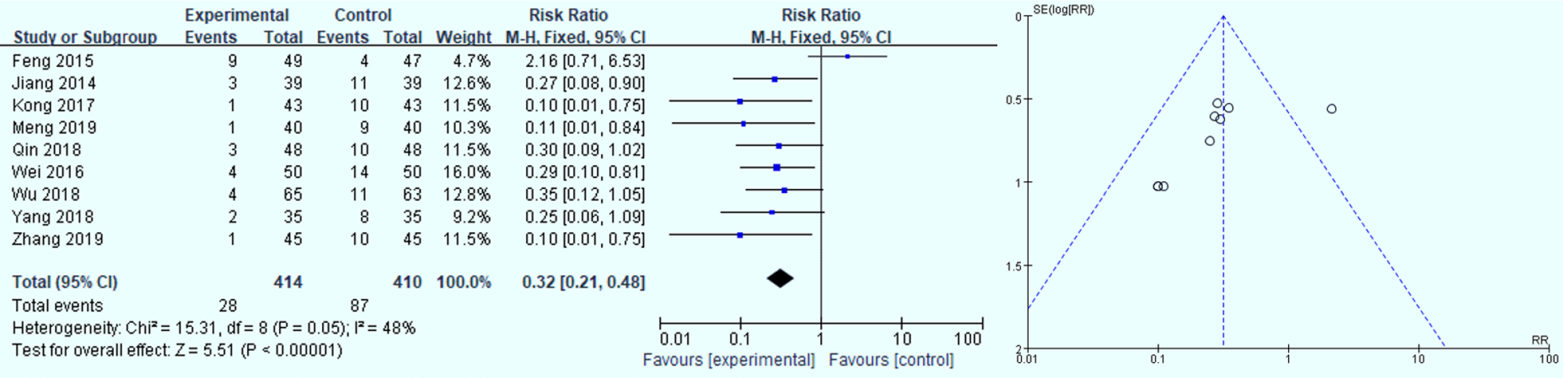
analysis of ineffectiveness of folic acid for GPC symptom relief

**Fig. 5:**
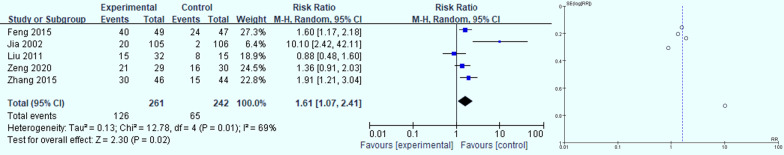
Reversal of gastric mucosal atrophy

Figure 3 summarizes the results of a meta-analysis of 9 studies on the influence of folic acid for GPC symptom [25–32, 34]. It can be seen from Fig. 4 that *P*=0.006, *I*^*2*^= 63%, indicating that the heterogeneity between studies is high, and the random effects model is used. The *RR* is 1.12 (*95% CI* *0.96-1.13, P*=*0.14*), The results are not statistically significant. Therefore, we made a comparative analysis of the ineffectiveness of folic acid treatment, and the results are shown in Fig. 5. A meta-analysis of the ineffectiveness of folic acid treatment through 9 studies [25–32, 34]. We found that *I*^*2*^ was 48%,*P*=0.05, indicating that the heterogeneity between the studies was low, and the fixed effects model was used. *P*<*0.00001,* the result is statistically significant. The *RR* was 0.32 (*95%CI 0.21-0.48*), which indicated that the treatment in the experimental group was 32% less effective than the control group. Thus, over the meta-analysis of folic acid treatment for GPC symptom effectiveness and ineffectiveness which included 2 studies. A comprehensive analysis can be drawn from the presence folic acid for the treatment of GPC may have effect. In addition, it was illustrated in the funnel chart that the risk of bias in the study is relatively large. So the certainty of the results needs to be further investigated.

#### Comparisons and outcomes2

Folic acid treatment of the pathological changes of GPC.

#### Outcomes 1

Changes in folic acid treatment of the gastric mucosa atrophy lesions.

The results of a meta-analysis of 5 studies [22,31,28,30, and an unpublished study written by Dr. Zeng] on the therapeutic effect of folic acid on pathological changes of gastric mucosa were shown. It can be seen from Fig. 5 that *P* = *0.01, I*^*2*^ = *69%*, indicating that the heterogeneity between the studies is high, and the random-effects model is used. The *RR* is 1.61*(95%CI 1.07–2.41, P* = *0.02)*, the result is statistically significant. The treatment of gastric mucosal atrophy with GPC can promote the change of gastric mucosal atrophy.

#### Outcomes 2

Changes in folic acid treatment of intestinal metaplasia lesions.

**Fig. 6:**
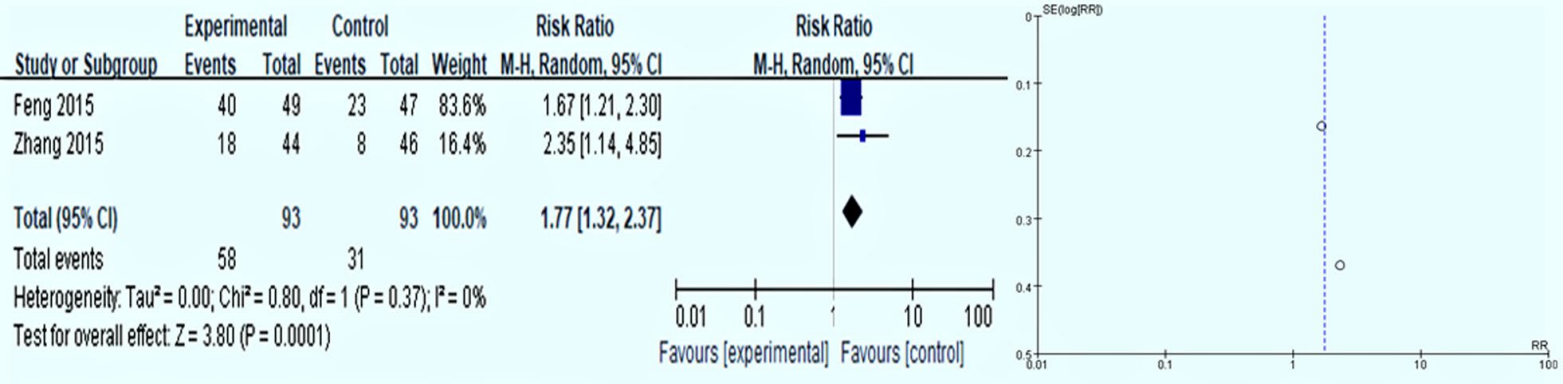
Intestinal metaplasia reversal

The results of 2 trails meta-analyses [27, 33] on the GPC of folic acid in the treatment of intestinal metaplasia. *P* = *0.37, I*^*2*^ = *0%*, indicating that there is homogeneity between the studies is high, and a random-effects model is used for analysis. The *RR* was 1.77 (*95%CI 1.32–2.37, P* = *0.0001)*, and the result is statistically significant. The results showed that compared with the control group, the effectiveness of folic acid in reversing the pathological changes of GPC intestinal metaplasia was 77% (Fig. 6).

#### Comparisons and outcomes3

Time-dose interval analysis.

**Fig. 7:**
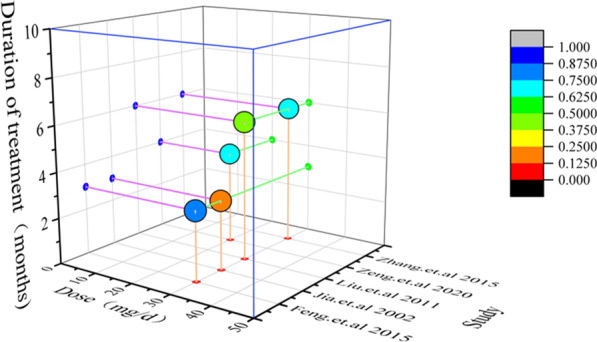
Time-dose interval analysis

Through time-dose interval analysis, we found that when the dose of folic acid was maintained at 20-30* mg / d* and the treatment duration was maintained at 3–6 months, folic acid had a significant therapeutic effect on the pathological changes of GPC. In addition, according to the analysis of 5 trails we also found that folic acid had a better therapeutic effect on the pathological changes of gastric mucosa of GPC when the dose was 30* mg / d* and the treatment duration was 3 months. This may suggest that the therapeutic effect of folic acid on the pathological changes of GPC may be positively correlated with the dose (Fig. 7).

## Discussion

Chronic atrophic gastritis is a common GPC, which is closely related to gastric cancer [1, 38]. Helicobacter pylori infection is an important pathogenic factor leading to the transformation from precancerous state to gastric cancer. It will cause the changes of pepsinogen and gastrin, thus increasing the risk of gastric cancer [39]. Pepsinogen is divided into 2 subgroups, PG I and PG II. When the gastric mucosa shrinks, the content of serum PG I decreases, but PG II remains relatively stable or slightly increased [40]. G cells in the antral mucosa and proximal duodenal mucosa secrete a hormone called gastrin. Its principal function is to stimulate parietal cells to secrete hydrochloric acid and to stimulate the secretion of bile and pancreatic juice (gastrin also weakly stimulates the pepsinogen-secreting chief cells). By detecting the levels of gastrin in the serum of patients with gastric cancer, Kang et al. [41] found that during sequential development of non-atrophic gastritis to gastric cancer, the production of gastrin gradually increased, and the G-17 increase in serum. The increase in G-17 content is oftentimes related to the occurrence of gastric cancer. Regarding risk factors, If G-17 is less than 1 pmol, it causes the parietal cells to secrete hydrochloric acid abnormally and reduces the absorptive and digestive functions of the stomach, the abnormal DNA methylation causes the gastric precancerous state to further deteriorate to the gastric precancerous lesions, and eventually evolve into gastric cancer. As a water-soluble vitamin, folic acid can act as an important methyl donor and participate in DNA synthesis and methylation, that folic acid may benefit for this condition.

A 7-year prospective, clinical cohort study in China [42] found that daily supplementation of 5–10 mg of folic acid for patients with GPC ameliorated histopathological abnormalities in the gastric mucosa. The research results [43] further show that lack of folic acid may result in gastric malignancy, and the supplementation of folic acid have prevent the further deterioration of gastric cancer. In contrast, Tang’s a double-blind experiment [39] showed that vitamin supplementation did not significantly improve the precancerous state of the stomach. However, some limitations, such as the amount of vitamins, the variability of biopsy and pathological tissue extraction readings, may reduce the reliability of research results. In addition, KIM and Zhu [44, 45] found that folic acid supplementation for patients over the age of 50 diagnosed with gastric cancer can promote the growth of gastric cancer cells and worsen their tumors.

Our review finally contained 13 studies with 1252 participants. We report the control and treatment effects of folic acid on GPC. The present study selected people in China who were diagnosed with chronic atrophic gastritis in accordance with clinical guidelines as researchers, and ruled out the risk of canceration to prove the effect of folic acid supplementation on the precancerous state of the stomach. In our study, although the result of the effectiveness of folic acid in the therapy of GPC symptom relief was not significant [25–32, 34], the analysis of the effectiveness of folic acid in the treatment of GPC symptoms showed that [25–32, 34], the effectiveness of folic acid in the treatment of GPC symptoms was as high as 68%. This indicates that folate has a positive influence on the treatment of GPC. In addition, a meta-analysis of the pathological changes after treatment in 2 comparison groups found that the meta-analysis results of 5 studies showed that folic acid can promote the recovery of gastric mucosal atrophic lesions [22,31,28,30,and an unpublished research written by Dr. Zeng. And 2 studies have shown that the effectiveness of folic acid treatment in reversing intestinal metaplasia in patients with GPC is 77% [27, 33]. In addition, according to our temporal dose analysis of 5 studies, when folic acid is administered at a dose of 30 mg* / d* for 1 month, the treatment will be better and more effective for the pathological changes of GPC. The above findings GPC is feasible to provide proof of folic acid treatment.

Besides, by comparing the changes in serum PG I, PG II, and G17 content before and after treatment of 2 comparison groups, we also confirmed that folic acid can inhibit development of gastric mucosal carcinogenesis by affecting the levels of gastrin and pepsinogen.

Our meta-analysis confirmed the preventive and therapeutic effects of folic acid on GPC, and provided further enlightenment for further popularizing the application range of fumarate. However, there were some limitations of the study. The age span and diet structure of the patients population have not been further distinguished. Although the time-dose interval analysis makes up for the deficiencies of the meta-analysis results, results should be interpreted with caution.

## Conclusions

Our study indicates that folic acid has a beneficial effect in the treatment of pathological changes of GPC when the dose was maintained at 20–30 mg*/d* and the duration of treatment was maintained at 3–6 months. Moreover there may be a direct correlation between the therapeutic efficacy and dose of folic acid on the pathological changes of GPC. And this may provide potential evidence for the clinical application of folate in GPC treatment.

## Supplementary Information

**Additional file 1:** Data collation:Raw data used in the study.

**Additional file 2: Table 2:** Description of included studies.

**Additional file 3: Table 1:** Baseline characteristics of study.

## Data Availability

The authors declare that [25–36] data supporting the findings of this study are available within the article. And the other data that support the findings of this study are available on request from the corresponding author [ZJH].

## Abbreviations

GPC: Gastric precancerous conditions
RR: Relative risks
PRISMA: Preferred reporting items for systematic reviews and meta-analyses
CI: Confidence intervals
RCT: Randomized controlled trials
